# Improving Mental Health in Pregnancy for Refugee Women: Protocol for the Implementation and Evaluation of a Screening Program in Melbourne, Australia

**DOI:** 10.2196/13271

**Published:** 2019-08-19

**Authors:** Jacqueline Anne Boyle, Suzanne Willey, Rebecca Blackmore, Christine East, Jacqueline McBride, Kylie Gray, Glenn Melvin, Rebecca Fradkin, Natahl Ball, Nicole Highet, Melanie Gibson-Helm

**Affiliations:** 1 Monash Centre for Health Research and Implementation School of Public Health and Preventive Medicine Monash University Melbourne Australia; 2 Monash Health Refugee Health and Wellbeing Monash Health Melbourne Australia; 3 Centre for Developmental Psychiatry & Psychology, Department of Psychiatry, School of Clinical Sciences Monash University Melbourne Australia; 4 Department of Obstetrics and Gynaecology Monash Health Melbourne Australia; 5 Monash Maternity Services Monash Health Melbourne Australia; 6 Centre of Perinatal Excellence Melbourne Australia

**Keywords:** mental health, refugees, transients and migrants, pregnancy, prenatal care, mass screening

## Abstract

**Background:**

Identifying mental health disorders in migrant and refugee women during pregnancy provides an opportunity for interventions that may benefit women and their families. Evidence suggests that perinatal mental health disorders impact mother-infant attachment at critical times, which can affect child development. Postnatal depression resulting in suicide is one of the leading causes of maternal mortality postpartum. Routine screening of perinatal mental health is recommended to improve the identification of depression and anxiety and to facilitate early management. However, screening is poorly implemented into routine practice. This study is the first to investigate routine screening for perinatal mental health in a maternity setting designed for refugee women. This study will determine whether symptoms of depression and anxiety are more likely to be detected by the screening program compared with routine care and will evaluate the screening program’s feasibility and acceptability to women and health care providers (HCPs).

**Objective:**

The objectives of this study are (1) to assess if refugee women are more likely to screen risk-positive for depression and anxiety than nonrefugee women, using the Edinburgh Postnatal Depression Scale (EPDS); (2) to assess if screening in pregnancy using the EPDS enables better detection of symptoms of depression and anxiety in refugee women than current routine care; (3) to determine if a screening program for perinatal mental health in a maternity setting designed for refugee women is acceptable to women; and (4) to evaluate the feasibility and acceptability of the perinatal mental health screening program from the perspective of HCPs (including the barriers and enablers to implementation).

**Methods:**

This study uses an internationally recommended screening measure, the EPDS, and a locally developed psychosocial questionnaire, both administered in early pregnancy and again in the third trimester. These measures have been translated into the most common languages used by the women attending the clinic and are administered via an electronic platform (iCOPE). This platform automatically calculates the EPDS score and generates reports for the HCP and woman. A total of 119 refugee women and 155 nonrefugee women have been recruited to evaluate the screening program’s ability to detect depression and anxiety symptoms and will be compared with 34 refugee women receiving routine care. A subsample of women will participate in a qualitative assessment of the screening program’s acceptability and feasibility. Health service staff have been recruited to evaluate the integration of screening into maternity care.

**Results:**

The recruitment is complete, and data collection and analysis are underway.

**Conclusions:**

It is anticipated that screening will increase the identification and management of depression and anxiety symptoms in pregnancy. New information will be generated on how to implement such a program in feasible and acceptable ways that will improve health outcomes for refugee women.

**International Registered Report Identifier (IRRID):**

DERR1-10.2196/13271

## Introduction

### Background

The perinatal period (from conception to 12 months following birth) is a time of increased vulnerability for the onset or recurrence of mental health disorders [1]. Perinatal depression and anxiety [2] affect up to 20% of all women and are recognized by the World Health Organization as major public health issues [3].

Perinatal mental health disorders have direct effects on women, their children, and families [4], including disrupted attachment between mother and infant [5] and elevated risk of maternal suicide. The latter is one of the leading causes of maternal death in high-income countries [6,7]. There is a substantial financial burden of maternal perinatal depression to individuals, private health insurance, governments, and the economy. For example, within the Australian economy, this was estimated at Aus $433.52 million in 2012 [8], and 8.1 billion pounds in the United Kingdom in 2014 [9]. Many factors contribute to a woman’s risk of developing perinatal mental health disorders. These include a history of mental health disorders, low socioeconomic status, intimate partner violence, isolation, previous trauma, and stressful life events [4,7,10-12].

Routine, standardized screening in pregnancy for mental health disorders is recommended in high-income countries, including the United Kingdom [13], the United States [14], and Australia [1]. Implementation of such processes requires consideration of each setting [15]. In Australia, these recommendations have not been well implemented because of significant barriers at both the level of the service and the individual. This represents a critical gap, and a lost opportunity, with women and families bearing the impact of missed diagnosis, early management, and support. Addressing these barriers is key to improving health care for women at increased risk.

### Refugee Women

The impact of the refugee experience on women cannot be underestimated. Women who are refugees have experienced one or more acts of violence related to war, persecution, gender-based violence, protracted situations of uncertainty for the future, and discrimination [7]. High rates of psychological disorders are evident and exacerbated by resettlement stressors such as language barriers, separation from or loss of family, cultural barriers, and marginalization [7,16,17]. The prevalence of mental health disorders in conflict-affected populations (men and women combined) is estimated to be 31% [18]. A recent systematic review of perinatal mental health of migrant women from low- and middle-income countries reported a pooled prevalence of 31% for any depressive disorder and 17% for a major depressive disorder [16]. Data specifically on mental health disorders in pregnancy for refugee women are lacking.

Screening may not be offered in routine care owing to a number of reasons: lack of validated screening tools in languages other than English; lack of interpreters; and lack of health professional skills and knowledge [1]. Previous research indicates that perinatal mental health screening is an acceptable practice in the maternity setting [19-21]. However, there is a paucity of published research focusing on women from culturally and linguistically diverse (CALD) backgrounds. To our knowledge, this is the first study that focuses on refugee women living in a resettlement and high-income country. Given the magnitude of the current global refugee crisis and migration patterns resulting in many CALD women living in high-income countries, our study will provide contemporary evidence on the acceptability and feasibility of perinatal mental health screening for this population.

### Context

#### Australia’s Multicultural Community

In 2016, Australia’s population was 23.4 million, of whom approximately 6 million (26%) were born overseas. Nearly 1 in 5 (18%) migrants have arrived since 2012, and over 300 separately identified languages are spoken in Australian homes [22]. In 2015, 25% of women who birthed in Australia were born overseas [23]. People arrive in Australia through 2 main migration programs: the migrant program for skilled workers and family migrants or the humanitarian program for refugees and those in refugee-like situations [24]. In 2017-2018, Australia’s total migration was 162,417, including 18,750 places allocated to the humanitarian stream [25,26].

#### Study Location

This study is being conducted in a large public health service in the southeast suburbs of Victoria’s capital city, Melbourne, in which perinatal mental health screening is not yet routine. It is one of Australia’s largest maternity providers and is located in a major area of refugee resettlement. The state of Victoria has the highest settlement of refugees in Australia, receiving approximately 33% of the national intake [27]. In the past 10 years (2008-2018), over 11,000 people from a refugee background have resettled in the southeast suburbs of Melbourne, the highest resettlement catchment in Victoria for refugees [28]. In addition, there are over 7000 people seeking asylum, who arrived without a valid visa, currently living in Victoria, representing about 40% of the national total [29]. Demographic trends for people of a refugee background show that most are aged under 35 years and approximately 50% are females [28]. Furthermore, this region of Melbourne is the most culturally diverse community in Australia, with residents from 157 birth places [30,31] and 45% to 60% of women who birthed were born overseas. It is one of the most socially disadvantaged areas in Australia, meaning many people are on the lowest quintile for access to material and social resources [32]. As no mental health screening currently takes place in pregnancy at this health service, it is expected that the prevalence and burden of diagnoses of depression and anxiety disorders in refugee women will be underestimated. Given the current understanding of the prevalence of such disorders among pregnant women generally and the refugee population specifically, this suggests a major gap in pregnancy care.

#### Australia’s Health Care System

Funded by the federal government, Medicare is Australia’s health care system which provides universal access to public hospital care, primary health care, and some allied health services [33]. Hospital care is free for a public patient at a public hospital with other services free or at a reduced cost. Eligibility for Medicare includes Australian citizenship, permanent residency, or having applied for permanent residency. A permanent protection visa, for people from a refugee background, also confers access to Medicare. For those seeking asylum, a number of factors, including Medicare eligibility, can influence access to universal health care. Successive Victorian state governments (where this study is based) have shown a commitment to optimizing health outcomes for people of a refugee background by investing in initiatives such as the Victorian Refugee Health Program and Refugee Fellow Program [34]. Furthermore, the Victorian Department of Health’s  *Guide to asylum seeker access to health and community services in Victoria 2011*, supports access to health care in a state-funded facility, regardless of Medicare status [35].

#### Pilot Work Informing the Program

Significant stakeholder engagement and formative research identified barriers and enablers to implementing a perinatal mental health screening program. Stakeholder engagement was undertaken across the state and included refugee health services, academics, community and hospital health services, and mental health and maternity health services. Interviews with 28 health care providers (HCPs) and 9 community representatives from diverse ethnic backgrounds identified a number of needs such as staff training in mental health screening and safety planning for women at risk, robust referral pathways, and translated versions of the Edinburgh Postnatal Depression Scale (EPDS) [36]. Community representatives identified additional factors such as awareness of mental health, appropriateness of tools, and availability of interpreters [36]. Importantly, this research reported strong support from the community and HCPs to undertake screening, identify women at risk, and provide early support and assistance [36]. On the basis of this formative research and in collaboration with the maternity and refugee health services in Southeast Melbourne, community women, nongovernment organizations, and academics, the co-designed screening program with refugee-appropriate referral pathways commenced in 2016.

### Screening Tools

#### The Edinburgh Postnatal Depression Scale

The EPDS is one of the most widely accepted screening measures for depression and anxiety symptoms in the perinatal period. It has been validated for use in pregnancy and the postpartum period [1] and has been validated in English as well as a number of other languages [37-39]. It is a 10-item, self-report questionnaire used to detect symptoms of emotional distress over the past 7 days [40]. The EPDS has been used internationally since its inception in 1987 and is available in many languages.

The English version of the EPDS performs with moderate sensitivity 0.83 (0.76-0.88) and high specificity 0.90 (0.88-0.92) in pregnancy [1]. The recommended cutoff score for use in general populations is 13 or above, indicating that depressive symptoms have been endorsed and signifying a high risk for probable depression which requires further clinical assessment. For women of CALD backgrounds, a lower cutoff score is recommended to balance psychometric performance with differences in cultural practices, beliefs, and degree of stigma [1]. Therefore, an EPDS cutoff score of ≥9 is used in this study, based on previous validations of EPDS translations with women of CALD backgrounds [41]. Although the EPDS was not designed to measure anxiety disorders, high scores on items 3, 4, and 5 have been found to be sensitive to symptoms of anxiety [42]. A score of ≥4 for the anxiety subscale is considered indicative of a high risk of anxiety symptoms and requires further assessment [42]. The final item (question 10) on the EPDS assesses the prevalence of suicidal ideation and risk of self-harm.

#### The Psychosocial Screening Tool

The Monash Health psychosocial needs assessment is a 23-item, locally developed, self-report measure specific to the health service that asks questions about risk factors for perinatal mental health disorders such as past birthing experiences causing stress or anxiety, social factors (such as housing and financial stress), experience of violence and safety at home, and a history of mental health disorders. In routine care, women complete the measure themselves or with the midwife at their booking visit. Respondents are required to provide “yes” or “no” answers and 4 nested text questions allow free-text responses.

For this study, the EPDS and the Monash Health psychosocial needs assessment have been translated to 7 refugee languages common in Southeast Melbourne: Arabic, Burmese, Dari, Farsi, Hazaragi, Pashto, and Tamil.

### Research Questions

The primary research questions are as follows:

1. Are refugee women more likely to screen risk-positive for depression and anxiety on the EPDS than nonrefugee women?
2. Does screening in pregnancy using the EPDS enable better detection of symptoms of depression and anxiety in refugee women compared with current routine care?

### Secondary Research Questions

We will also explore the following secondary questions:

1. Is perinatal mental health screening in pregnancy using an electronic platform acceptable and feasible to refugee women?
2. What are the barriers and enablers to the screening being perceived as a feasible and acceptable part of the routine practice by maternity HCPs?

### Hypotheses

We hypothesize that a perinatal mental health screening program that addresses key concerns of women and HCPs can improve identification of symptoms of perinatal depression and anxiety in refugee women. We also hypothesize that co-designed screening can be implemented within a large and busy maternity service in a manner that is acceptable to both women and health service staff.

## Methods

### Setting

The study is being conducted at a refugee antenatal clinic (RAC) designed for refugee women in Southeast Melbourne, Australia. This clinic operates 1 day per week and is supported by a refugee health nurse liaison (RHNL) and 2 bicultural workers. On receipt of a general practitioner (GP) referral for maternity care, all women are allocated, by hospital clerical staff, to either the RAC or one of the other antenatal clinics on the basis of availability and preference. Therefore, refugee women also attend the other maternity clinics at the health service and nonrefugee women attend the RAC. On average, 13 women attend their first appointment with a midwife each week at the RAC. Approximately half of the women attending are from a refugee background or considered refugee-like, that is, arrived in Australia on a spousal visa from a refugee-source country, including Afghanistan, Myanmar, Iraq, the Republic of South Sudan, and Sri Lanka.

### Procedures

#### Ethics Approval

This project has been approved by the Monash Health Human Research and Ethics Committee number 14475L.

#### Participants and Recruitment

The day before the first appointment, a female Afghan bicultural worker (RA) or one of the researchers (RB) telephones women to remind them of their appointment and to explain the screening and recruitment process for the research. Interpreters are used for women who do not speak the same language as RA or RB. Researchers are present at the clinic on the day of the appointment and obtain written informed consent from each participating woman. Consent is requested to access data from their medical health records at the hospital, GP records, and Monash Health Refugee Health and Wellbeing (RH&W) service records. This will enable evaluation of the screening results, referrals, and subsequent diagnosis and management. Women are also invited to participate in the acceptability phase of the project.

All staff working in the RAC (clerical staff, midwifery, medical, bicultural workers, and RHNL) and at the RH&W (psychologists and counselors) are invited to participate in the evaluation of the feasibility of the program.

#### Intervention

On the day of the first appointment, all women attending the RAC are given an iPad to complete the screening (EPDS and Monash Health psychosocial needs assessment) using the digital platform iCOPE. iCOPE has been developed and piloted by the Centre of Perinatal Excellence (COPE, Australia) [43]. Women are able to complete the screening in their chosen language in the clinic waiting room and interpreters or bicultural workers are available to assist. Screening is repeated in the third trimester. It takes approximately 6 to 10 min for a woman to complete the screening on her own, or slightly longer if an interpreter is used. The iCOPE platform automatically calculates the overall EPDS score, the anxiety subscore (based on responses to question 3, 4, and 5), and highlights the response to question 10, which assesses risk of self-harm. Data are securely stored in compliance with industry regulations [43].

#### Co-Designed Referral Pathways for Refugee Women

**Figure 1:**
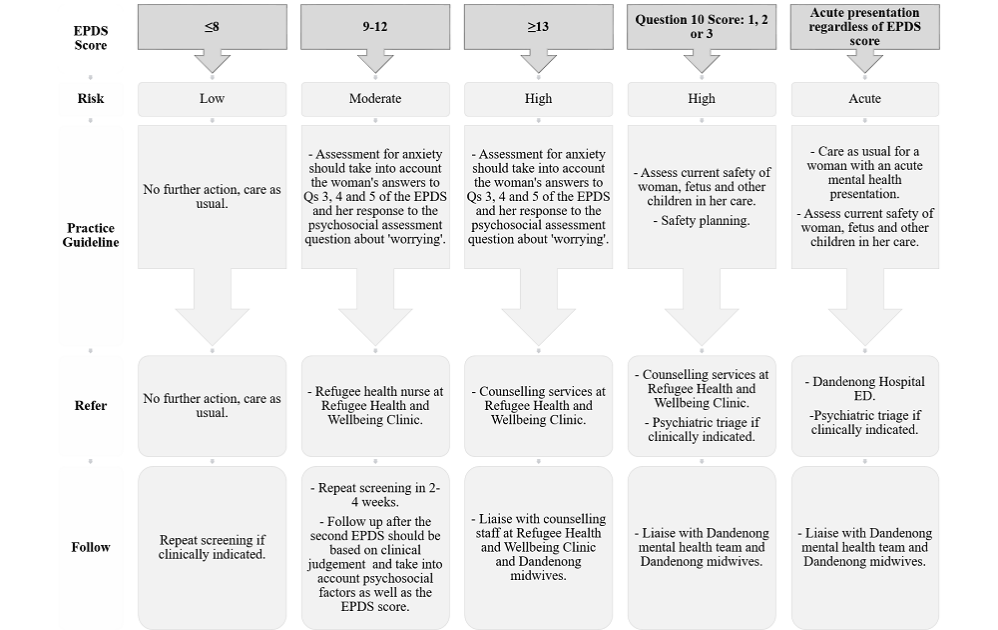
Referral pathway for women of refugee background. ED: emergency deparmtent; EPDS: Edinburgh Postnatal Depression Scale.

During the appointment, the midwife discusses the result with the woman and initiates referral as appropriate. If the overall EPDS score is ≥9, the score for the anxiety subscale is ≥4, or the response to question 10 (self-harm) is positive, the RHNL is notified and further mental health and psychosocial assessment is undertaken. If the assessment by the RHNL indicates the woman is acutely ill, at risk of harming herself or others, an immediate referral to the hospital emergency department “and” or “or” psychiatric services is made. If the woman is not acutely unwell, the RHNL will refer to the RH&W counseling or to their GP, if preferred. If the result is between 9 to 12, a repeat screen in 2 to 4 weeks is recommended (see referral pathways, Figure 1). On completion of screening, women are provided a report in their chosen language that explains their results and a link to further resources and supports via email. A clinical report and management guide is also immediately generated for the midwifery appointment. If other factors are present, such as housing concerns or intimate partner violence, appropriate referrals are made as per usual care to services such as social work or legal services.

#### Comparison Group 1: Nonrefugee Women Attending the Refugee Antenatal Clinic

**Figure 2:**
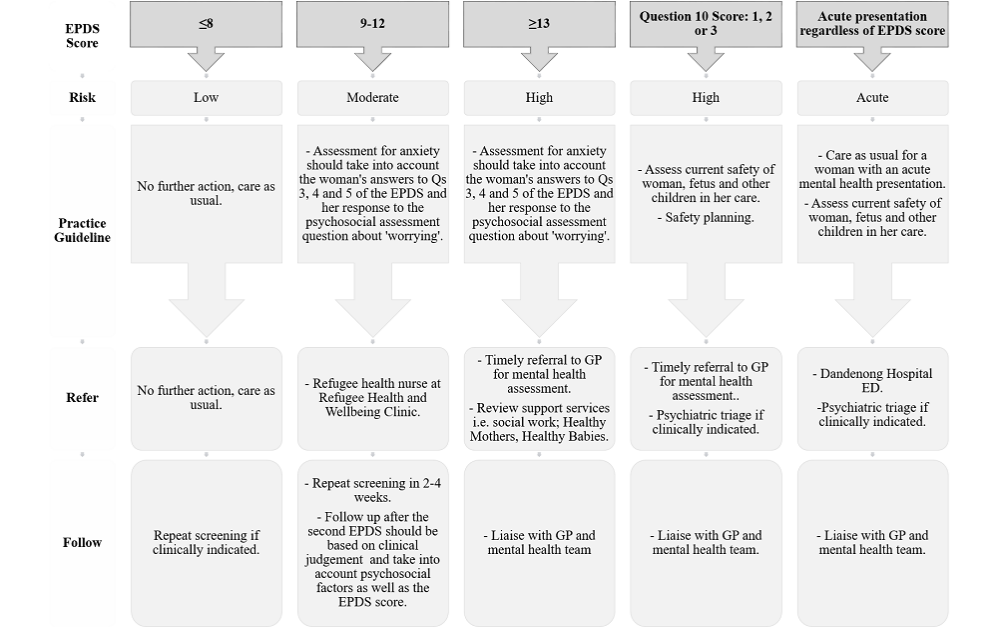
Referral pathways for women of nonrefugee background. ED: emergency department; EPDS: Edinburgh Postnatal Depression Scale; GP: general practitioner.

Nonrefugee women who attend the RAC also complete screening of the EPDS and the Monash Health psychosocial needs assessment using the iCOPE platform. Referral options include referral to the hospital emergency or psychiatric services if women are acutely unwell. For others, options include repeat screening in 2 weeks, allied health support such as social work, and referral to the woman's GP (see referral pathways for nonrefugee women, Figure 2).

#### Comparison Group 2: Refugee Women Attending Other Routine Antenatal Services at the Same Hospital

Refugee women also attend other antenatal clinics at the health service. Routine care at these clinics include completing a paper-based Monash Health psychosocial needs assessment (no EPDS) with the midwife during the first antenatal appointment. Routine referral pathways are not proscriptive but may include the RHNL, GPs, and services such as social work.

### Evaluation of the Perinatal Mental Health Screening Program

#### Research Questions 1 and 2

Medical records will be audited for women attending the RAC and participating in the research program (119 refugee women and 155 nonrefugee women) and for a random sample of refugee women attending routine maternity clinics (non-RAC) during the study period (n=34). Information collected will include demographic data (including factors such as age, country of birth, time since arrival in Australia, marital status, number of pregnancies and births, and need for an interpreter), medical history (eg, diabetes, hypertension, and smoking), and psychosocial needs assessment results.

For those attending the RAC, the additional information of the screening report, EPDS scores (total, anxiety subscore, and Question 10 score) are recorded.

For the refugee women attending the non-RAC clinic, who do not participate in the screening program, any diagnosis of past or present mental health disorders and any relevant referral or management are recorded.

Data will be deidentified and entered into a REDCap database (Vanderbuilt, USA) [44] for collation and analysis. To address research question 1, the proportion of refugee women who screen positive on the EPDS for depression and anxiety will be compared with the proportion of nonrefugee women who screen positive.

To address research question 2, the proportion of refugee women who screen positive to depression or anxiety on the EPDS will be compared with the proportion of nonrefugee women attending routine maternity clinics who are identified with mental health disorders.

#### Research Question 3

To evaluate the women’s acceptability of the screening program, women from the majority population groups at the RAC (refugee: Afghan and Burmese; nonrefugee: Indian and Vietnamese) are invited to participate in either a focus group or an interview to discuss their experiences of screening and referral. Interpreters will be engaged to maximize inclusion. Focus groups “and” or “or” interviews will continue until data saturation of themes is achieved.

#### Research Question 4

The Normalization Process Theory to Assess Health Care Providers’ Views on Implementation:

All HCPs and clerical staff involved in the screening program (at the RAC and the RH&W) are invited to participate in an evaluation of implementation processes. This includes completion of the 23-item Normalization Measure And Development (NoMAD) online survey adapted for this study and participation in an interactive focus group or interview using the Normalization Process Theory (NPT) toolkit. The NoMAD was distributed through Qualtrics (Provo, Utah, USA). The NPT toolkit has been selected and adapted for this project [45] as it focuses on implementation and assesses 4 relevant constructs: (1) coherence (how do staff make sense of the program when operationalizing the new set of practices), (2) cognitive participation (what work are the staff required to do to build and sustain a community of practice around the program), (3) collective action (what operational work is required by the staff to enact the new practice), and (4) reflexive monitoring (appraisal by the staff in understanding how this new set of practices affects them and others around them) [45].

### Medical Records Audit

#### Women Attending the Refugee Antenatal Clinic

The medical records audit already described notes referrals to primary care, allied health, counseling, psychiatry, emergency services, or others, within and outside the hospital, and referrals made following screening with the EPDS and psychosocial needs assessment or in response to other clinical indications. The number of women who attend appointments arising from these referrals within the hospital or at the RH&W will be recorded. When a referral has been made to a GP (external to the health service), the GP practice is contacted to ascertain whether the woman attended for a formal assessment and diagnosis, the outcome, and any subsequent management plan.

#### Women Attending the Nonrefugee Antenatal Clinic

Similar data are collected about referrals made based on clinical assessment of need and subsequent attendance.

### Outcome Measures

#### Primary Outcomes

The primary outcome is the proportion of women in 3 groups (refugee women screened, nonrefugee women screened, and refugee women receiving routine care) with symptoms of depression and/or anxiety.

#### Secondary Outcomes

Secondary outcomes include identification of factors that will influence broader implementation of screening:

- Factors that facilitate acceptability of the program to women
- Factors impacting positively and negatively on the feasibility of program implementation at a systems level.

### Analysis Strategy (Sample Size Justification)

#### Research Question 1: Are Refugee Women More Likely to Screen Risk-Positive for Depression and Anxiety on the Edinburgh Postnatal Depression Scale Than Nonrefugee Women?

It is estimated that 40% of refugee women [16] and 20% of nonrefugee women will have an overall EPDS score of ≥9 [1]. The number of women required to detect a difference of 20% between the 2 groups, with 90% power, is 119 per group.

Additional analyses will assess differences in the proportion who score positive for anxiety or at-risk on question 10 of the EPDS.

#### Research Question 2: Does Screening in Pregnancy Using the Edinburgh Postnatal Depression Scale Enable Better Detection of Symptoms of Depression and Anxiety in Refugee Women Compared With Current Routine Care?

It is estimated that 40% of refugee women will have an overall EPDS score of ≥9 [7,16,46]. Current health service data indicate that less than 5% of women attending routine maternity care are recorded as having a mental health disorder. The number of women required to detect a difference of 20% between the 2 groups, with 80% power, is 34 per group.

#### Statistical Analysis for Research Questions 1 and 2

Data will be assessed using Stata Statistical Software: Release 14 (StataCorp, College Station, TX, US) [47] and will use chi-square tests for proportions, Student *t* test for comparisons of means, Wilcoxon rank sum tests for comparison of medians, and paired *t* test to compare the EPDS and anxiety subscores between initial and third trimester screening. Univariable and multivariable logistic regression analyses will be used to assess the impact of demographic factors such as marital status, country of birth, time since arrival in country, age, and parity on the primary outcome (overall EPDS score of ≥9).

#### Research Question 3: Is Perinatal Mental Health Screening in Pregnancy Using an Electronic Platform Acceptable and Feasible to Refugee Women?

Qualitative data will undergo thematic analysis to enable in-depth exploration of the data. Interviews will be audio recorded and transcribed verbatim. Transcripts will be read several times to obtain a sense of the whole before analysis. Overall, 2 researchers will independently conduct the initial narrative analysis using NVivo 11 (QSR International, Australia) qualitative data analysis software [48]. In the second phase, pieces of the data conveying the situation, the experiences, and the beliefs of participants will be identified and highlighted. A third phase involves the data being organized into patterns and emerging categories. Finally, a process of synthesis of the data will be undertaken that will result in the identification of major themes [49,50].

#### Research Question 4: What Are the Barriers and Enablers to the Screening Being Perceived as a Feasible and Acceptable Part of the Routine Practice by Maternity Health Care Providers?

A similar process will be undertaken with the NPT-based interview and focus group transcripts with the HCPs. Separate analysis of the NoMAD quantitative data (online survey) will be undertaken to assess responses and assess any differences by factors such as HCP type, age, and years of practice. The qualitative and quantitative data will then be combined, and mixed-methods analytic techniques will be applied [50]. Merging and connecting data and finally interpreting the data enables the researcher to draw inferences on the overall mixed-methods analysis [50].

## Results

Recruitment of 119 refugee women and 155 nonrefugee women is complete. Data collection and analysis are underway. The cohort reflects the multicultural aspects of the community, with 248 of 274 (90.5%) women born overseas and 190 of 274 (69.3%) women arriving in Australia between 2008 and 2017.

## Discussion

Stakeholder engagement and governance are key components of this research program. This ongoing stakeholder involvement has enabled the program to be co-designed and to evolve to meet stakeholder needs. The steering committee comprises staff from key hospital departments, GP liaison, RH&W, the nongovernment organization COPE, and academic experts in psychology, midwifery, obstetrics, and public health. This committee has met fortnightly for 2 years to plan, implement, and evaluate the program. The committee addresses concerns of the research team or hospital staff as they arise and responds with practical solutions. A community advisory group comprising women from 8 different countries also meets bimonthly and has been instrumental in planning the implementation and evaluation such as recruitment strategies, resources, and facilitating an understanding of the cultural complexity of the women participating.

## Abbreviations

CALD: culturally and linguistically diverse
COPE: Centre of Perinatal Excellence
EPDS: Edinburgh Postnatal Depression Scale
GP: general practitioner
HCP: health care provider
NoMAD: Normalization Measure And Development
NPT: Normalization Process Theory
RAC: refugee antenatal clinic
RH&W: Monash Health Refugee Health and Wellbeing
RHNL: refugee health nurse liaison
